# Water and Filth: Reevaluating the First Era of Sanitary Typhoid Intervention (1840–1940)

**DOI:** 10.1093/cid/ciz610

**Published:** 2019-10-15

**Authors:** Samantha Vanderslott, Maile T Phillips, Virginia E Pitzer, Claas Kirchhelle

**Affiliations:** 1 Oxford Vaccine Group/Oxford Martin School, University of Oxford, United Kingdom; 2 Department of Epidemiology of Microbial Diseases, Yale School of Public Health, Yale University, New Haven, Connecticut; 3 Wellcome Unit for the History of Medicine/Oxford Martin School, University of Oxford, United Kingdom

**Keywords:** typhoid control, sanitary reform, public health history, typhoid mortality

## Abstract

While typhoid fever remains an important cause of illness in many low- and middle-income countries, important insights can be learned by exploring the historical experience with typhoid fever in industrialized countries. We used archival research to examine British and American attempts to control typhoid via sanitary interventions from the 1840s to 1940s. First, we assess how varying perceptions of typhoid and conflicts of interest led to a nonlinear evolution of control attempts in Oxford, United Kingdom. Our qualitative analysis shows how professional rivalries and tensions between Oxford’s university and citizens (“gown and town”), as well as competing theories of typhoid proliferation stalled sanitary reform until the provision of cheap external credit created cross-party alliances at the municipal level. Second, we use historical mortality data to evaluate and quantify the impact of individual sanitary measures on typhoid transmission in major US cities. Together a historiographic and epidemiological study of past interventions provides insights for the planning of future sanitary programs.

Popular accounts of Western typhoid control often follow heroic narratives of a “great sanitary awakening” [1] with a deceptively clean chronology of typhoid’s pathological distinction from typhus (William Gerhard, 1836) [2], proof of typhoid’s transmissibility via water (William Budd, 1856 & 1873) [3], *Salmonella* Typhi’s isolation and proof of pathogenicity (Karl Eberth, 1880; Georg Gaffky, 1884) [4, 5], the development of the Widal serological agglutination test for typhoid, and the discovery of (asymptomatic) typhoid carriers (Robert Koch, 1902) [6].

Progress was supposedly inevitable once “rational” practitioners had discovered typhoid’s microbial cause and debunked competing theories. While we do not discount the role of the contemporary discovery of typhoid’s waterborne transmission and bacterial causation, historians have shown that narratives of a resulting linear evolution of successful science-informed control measures are inaccurate: Clinical definitions of typhoid remained in flux for most of the 19th century; pathologies were not routinely conducted and did not always provide clear results; the bacteriological revolution filtered unevenly through society; serological diagnosis remained error-prone; and *Salmonella enterica* serovar Typhi taxonomies remained contested until the 1930s. Despite typhoid fever’s prominence in contemporary discourse and statistics, extrapolating disease incidence from historical sources is thus ridden with difficulties. There was also no inevitable correlation between growing awareness of typhoid and the implementation of effective sanitary control strategies. Meanwhile, historians continue to disagree on sanitary interventions’ relative impact on typhoid mortality in relation to other factors such as improved healthcare and nutrition [7–10].

Here, we reevaluate the history of typhoid control in Britain and the United States between the 1840s and 1940s. During this period, both countries undertook significant sanitary interventions to control enteric infection. Using historical and epidemiological methodologies, we examine different interventions’ origins and impacts. First, we examine the hit-and-miss evolution of 19th-century sanitary interventions. The city of Oxford (United Kingdom) is exemplary of the impact of changing central government powers alongside changing concepts of typhoid transmission and causation. Second, we examine the various approaches to improving water and sanitation infrastructure across different representative cities in the United States around 1900. Our analysis highlights how the availability of cheap municipal credit played an important role in bringing together important actors to fund the infrastructure development necessary for typhoid control.

## OXFORD, A CONTAGIOUS CITY

Oxford’s environmental context, mixed urban-rural structure, rapid expansion, and pronounced social tensions make it a useful case study that can also provide insights for current typhoid control strategies in rapidly urbanising contexts. Historically, Oxford shared many of the socioenvironmental features exhibited by present-day typhoid-endemic settings. Environmentally, the city’s low-lying location at the confluence of 2 rivers and porous soil structure provided conditions for typhoid proliferation as a result of flooding and the contamination of drinking water wells with human waste [11]. Structurally, the city boundaries comprised both high-density urban and rural areas as a result of rapid population growth but comparatively slow industrialization [12]. Socioeconomically, life in Oxford was characterized by pronounced divisions between an upper-class mostly university-affiliated elite and the remainder of the town’s middle- and lower-class citizens [13]. We used archival research in local government and University of Oxford collections to elucidate the conditions and factors that ultimately led to the implementation of typhoid control strategies in Oxford.

According to contemporaries, sanitary conditions in early 19th-century Oxford were dire. In 1848, the Health of Towns Association reported that the municipal water supply was “intermittent and very deficient”: Intakes were at “the lowest level of the city, and at the tail of nearly all the sewers” [14], and high prices meant that only 3.56% of city dwellings were supplied by piped water [15]. Remaining citizens either drew water from often contaminated wells or directly from the city’s rivers.

Problematic sanitary arrangements came under pressure from the mid-19th century onward. Trained in statistical analysis and mobilizing the spectres of cholera and typhoid, a new generation of young politically engaged experts pushed for social and sanitary reform [16]. In Oxford, the reform movement was spearheaded by enterprising university surgeons and physicians based at the Radcliffe Infirmary. Between 1848 and 1854, William P. Ormerod, Thomas Allen, William Alexander Greenhill, and Henry Wentworth Acland (later Regius Professor) compiled mortality statistics and epidemiological maps of the 3 cholera waves hitting Oxford in 1832, 1849, and 1854 as well as of noncholeraic “enteric” fevers. Their findings highlighted higher mortality in Oxford’s low-lying and overcrowded working-class parishes. Mixing miasmatic (“bad air”) and new waterborne concepts of enteric disease transmission, they urged municipal authorities to tackle odiferous filth and stagnant water by improving drainage, sewerage, and utilizing the 1848 Public Health Act, which had established a General Board of Health capable of providing or authorising cheap loans for infrastructure [17–20].

Sanitarians’ reform proposals proved controversial: While the University’s commissioners supported the proposals, many town representatives opposed sanitarians’ plans, which the University refused to pay for and which would entail a loss of local autonomy and more central government interference [21]. There was also significant contemporary disagreement about sanitarians’ theories of enteric disease transmission. In contrast to sanitarians’ focus on preventing flooding and improving drainage, local opponents claimed that frequent flooding improved health by removing odiferous matter whereas others claimed that cholera was caused by a fungus or alluvial soil and had nothing to do with water [22]. Although contemporary work by William Budd was proving that typhoid transmission was primarily waterborne [3], this knowledge did not play an authoritative role in Oxford’s early sanitary reform debates.

Between 1849 and the 1860s, town and gown disagreements on the cause of Oxford’s disease problems and who should pay for removing them led to a wave of commissioned external sanitary assessments. Often conducted by civic engineers, whose professional interests closely aligned with those of sanitary reformers, resulting reports stressed the need for improved sanitation to remove putrefying and odiferous matter causing miasma and proposed various new sewerage infrastructures. However, ongoing power struggles meant that all were turned down for reasons of cost and disagreement on who should pay for change.

While many of the city’s early engineering schemes focused on removing miasmatic causes of disease via sewage and drainage, Oxford’s drinking water also remained problematic. Although it stopped directly pumping drinking water from below sewage outlets in 1853, the city ignored advice for new waterworks to be situated upstream of Oxford and instead acquired cheap low-lying spring-fed gravel pits (formerly used for railway construction) close to its former intake and the cesspits of the rapidly expanding Hincksey neighborhood. The new water source was occasionally topped up with river water and could also be contaminated during floods. Despite improved pumping, simple gravel and sand filtration, and new cast iron pipes, municipal water’s cost and poor quality made the majority of Oxford’s poorer classes continue to favor wells and river water while University colleges benefited from slightly better arrangements with piped water being supplied from a higher spring on Hincksey Hill [15]. With Oxford divided in terms of its expertise, town and gown populations, and water infrastructure, enteric disease remained rife.

The sanitary stalemate was only broken around 1870 by university typhoid outbreaks, central government pressure, and the availability of cheap credit [11]. Between 1850 and 1870, a burgeoning population placed additional pressure on Oxford’s already weak water infrastructure. Oxford’s growth also flattened the spatial segregation between town and gown—with poorer students allowed to live in nonuniversity lodging houses from the 1860s onward. Resulting sanitary problems—in particular typhoid outbreaks—soon attracted national attention. In 1871, Britain’s Chief Medical Officer, Sir John Simon, publicly complained about the unsatisfactory state of public health and sanitary arrangements in Oxford [11]. Three years later, the death of 3 of 4 undergraduates who had contracted typhoid in Oxford lodging houses made national news. In January 1875, *The Lancet* proclaimed: “There can be no doubt that at the present moment Oxford is not a safe place of residence, owing to the imperfect condition of its drainage and impure water supply” [23]. It was also claimed that Prince Leopold, Queen Victoria’s youngest son, had contracted typhoid while living in Oxford as an undergraduate [24, 25].

**Figure 1.**
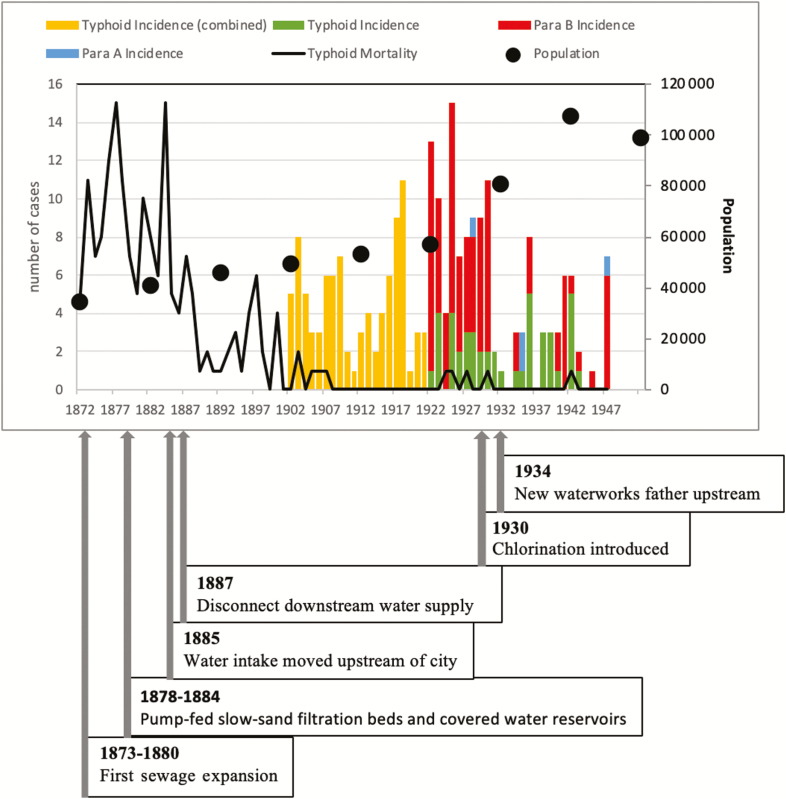
Historical typhoid mortality and morbidity data in Oxford, United Kingdom. Source: Oxford Medical Officer of Health Annual Reports, Oxfordshire History Center.

While frequent typhoid outbreaks in Oxford’s working-class districts rarely attracted much attention, cases among the student body reflected badly on both the University and senior municipal officials. At first, the University tried to assuage public criticism with stricter inspections by its commission for nonuniversity lodging houses (est. 1868) [11]. However, cheap external money also created a pact for more systematic sanitary reform between town and gown. From the 1870s onward, a combination of cheap government loans with often very long periods of repayment (in some cases over a century) and sanctioned credits led to a boom of municipal investment in sanitary and water infrastructure across England [26, 27]. In Oxford, municipal credit solved previous disagreements about who should foot the bill and harmonized sanitary campaigning by university representatives like Henry Acland and officials like Alfred Winkfield and Gilbert Child, Oxford’s and Oxfordshire’s first public health officers, and William Henry White, Oxford’s chief engineer [28]. Between 1873 and 1877, Oxford rapidly expanded its sewerage system, systematically replaced privies with flushing water closets, stopped piping human waste into the Thames, and began to pump effluent onto a municipal sewage farm. Using new funds resulting from a Waterworks Bill to Parliament, the city also improved its water supply. Between 1878 and 1885, it installed new pump-fed slow-sand filtration beds and a higher-lying covered fresh water reservoir for filtered water. Filtering beds began to be used from June 1885. It was also decided to move Oxford’s main supplemental water intake upstream of the city and disconnect direct intakes of river water that had already flowed through the city, which was achieved in October 1887. By 1886, many of Oxford’s houses were connected to a now financially viable rate-financed municipal water supply—thereby ending separate town and gown systems [15, 29, 30]. With contemporary experts only slowly acknowledging new bacteriological theories of typhoid and other diseases [10, 31, 32] a further expansion of the sewerage system to encompass poorer districts like Osney and St Clement’s took place between 1884 and 1920 and the original waterworks were abandoned in favor of new pumped, filtrated, and chlorinated (1930) supplies from further upstream in 1934 (Figure 1) [15].

The long-term effects on typhoid incidence were pronounced. While diagnostic problems make misreporting and underreporting likely, contemporary data indicate a decline of typhoid mortality from a high of 15 cases (1878 and 1884) to virtually zero between 1908 and 1915, with particularly sharp dips occurring between the installation of improved sand filtration, new upstream water intakes, and the universal provision of municipal piped water (1883–1886). Data on typhoid incidence (available from 1898 onward) indicate a further sharp decline following the establishment of chlorination (1930) and the closure of the old water works (1934) (Figure 1). In keeping with national trends, municipal expenditure on the maintenance and expansion of water and sewage infrastructure as well as on slum clearance continued to expand throughout this period [26, 27]. By the late 1930s, >6 decades of debt-fueled and conflict-ridden sanitary reform had dispelled Oxford’s contagious reputation.

Historically, new scientific knowledge of waterborne transmission and bacterial causation thus played an important role in gradually changing local understandings of typhoid but was not the sole factor shaping the trajectory of municipal disease control measures. Instead, the combination of cheap municipal credit, scandals, and external pressure proved crucial in breaking down old rivalries around sanitary reform—be it to remove miasmatic filth or dangerous bacteria—and creating the financial capacity for a sustained program of municipal sewage and water systems expansion.

## INFRASTRUCTURE INVESTMENTS AND DECLINING TYPHOID MORTALITY IN US CITIES

Nonlinear responses to local disease problems also occurred in cities across the United States. Similar to Oxford, local experts frequently presented sanitation as a major cause for typhoid reductions, as well as reductions in overall mortality [33]. However, the varying nature of local interventions makes this claim difficult to assess [34]. Here we examine the evolution of municipal sanitation strategies, investments in water and sewer systems, and their impact on typhoid transmission, as illustrated by the experience in 4 representative cities with different water supply sources.

Despite being influenced by British developments, American sanitary reform had a unique trajectory. Similar to Britain, the germ theory of disease was still hotly debated among US physicians and sanitary reformers prior to the mid-1880s [35]. Nevertheless, sanitary reformers were united in their efforts to build sewers and remove “filth” during the latter half of the 19th century, but urban development stalled during the Civil War of the 1860s [36]. Around 1900, US typhoid control was thus deemed to lag behind European efforts [8]. Waterborne diseases were responsible for almost 25% of reported deaths from infectious diseases in major US cities, which were experiencing rapid immigration, industrialization, and overcrowding [37]. Similar to Oxford, American city dwellers washed waste into sewers, which then emptied back into rivers and lakes that served as municipal water supplies. Effective hygiene, sanitation, and clean water systems were limited. As a result, outbreaks of infectious diseases, including typhoid, were regularly reported in larger cities [26, 38].

Until the Second World War, US legal requirements for water disposal and treatment varied significantly [39]. Beginning with Massachusetts in 1869, state boards of health were largely responsible for ensuring water quality. Coverage was patchy, and a 1905 survey identified only 36 states with any laws protecting drinking water [40]. In 1914, the newly founded Public Health Service organization created the earliest form of national drinking water guidelines to prevent infectious disease transmission. However, it was not until 1948 that federal laws were enacted and enforced to establish national treatment and drinking water standards, culminating in the Clean Water Act (1972) and Safe Drinking Water Act (1974) [41].

The absence of national standards led to different municipal sanitation strategies. While Philadelphia built the first large-scale municipal water system in 1802 [37], there was no effective method for waste removal until the 1850s. Following the British model, many US cities began to construct sewage systems during the 1880s [40]. However, despite the growing consensus on the waterborne mode of enteric disease transmission, early water supply and sewer systems were not necessarily effective at preventing infectious diseases [37]. With often contaminated public water supplies proving popular among poorer segments of the population, the lack of effective treatment could initially have further spread disease. In 1872, Poughkeepsie, New York was the first city to adopt slow-sand filtration to its water supply system [42], but the new technology was expensive and slow to spread to other cities. Responding to new bacteriological concepts of typhoid, Jersey City was the first US city to employ large-scale use of chlorination in 1908, which was both an effective and inexpensive way to purify the water supply [43] and was soon adopted by other US cities.

To test claims that municipal investment in water and sewer systems was associated with declining typhoid mortality [37], we extracted city-level weekly typhoid mortality data from 1889 to 1931 from the Project Tycho database (Table 1) [43, 44]. The database is based on digitized weekly morbidity and mortality reports, which the US government began to compile nationally alongside demographic data from 1888 onward [45]. Demographic and financial data on water supply and sewer systems for each city were obtained from annual US Census Bureau reports [46, 47]. Here, we focus on the experience in 4 cities: Philadelphia, New York, Chicago, and San Francisco; analysis of data from additional cities has been reported previously [48]. Together, these 4 cities reported 46 427 typhoid deaths over the 4 decades. While the typhoid mortality data remain marred by the same biases, gaps, and inaccuracies as in Oxford [44, 45, 49], the consistency of reporting over time allows for an analysis of long-term trends.

**Table 1.** Descriptive Statistics of Cities and Their Water Supplies

City	State	Total No. of Typhoid Deaths^a^	Population in 1888	Year of Filtration Introduction	Year of Chlorination Introduction	Year and Type of Other Clean Water Technology Introduction
Baltimore	MD	5198	431 000	1914	1910	
Boston	MA	3412	414 000	…	…	1908: New reservoir
Chicago	IL	13 161	981 000	1900	1912	1900: Changed river flow
Cincinnati	OH	3292	289 000	1908	1911	
Cleveland	OH	3622	241 000	1917	1913	
Milwaukee	WI	1912	187 000	…	1910	
Nashville	TN	1535	69 594	1908	1909	
New Orleans	LA	3352	237 000	1900	…	1900: Drainage
New York	NY	16 991	2 370 000	1903	1912	1905–1915: New reservoirs
Philadelphia	PA	13 927	1 010 000	1902	1913	
Pittsburgh	PA	7864	322 000	1908	1910	
Providence	RI	1106	127 000	1902	…	
Saint Louis	MO	3271	432 000	1904	1912	
San Francisco	CA	2348	286 000	…	…	1906: Earthquake and fire^b^
Toledo	OH	1381	75 167	1910	1910	
Washington, DC	…	3651	214 000	1903	1923	

Abbreviations: CA, California; DC, District of Columbia; IL, Illinois; LA, Louisiana; MA, Massachusetts; MD, Maryland; MO, Missouri; NY, New York; OH, Ohio; PA, Pennsylvania; RI, Rhode Island; TN, Tennessee; WI, Wisconsin.

^a^Number reported after imputation for missing data.

^b^No interventions were identified for San Francisco, but the 1906 earthquake resulted in necessary rebuilding of water and sanitation infrastructure.

**Figure 2.**
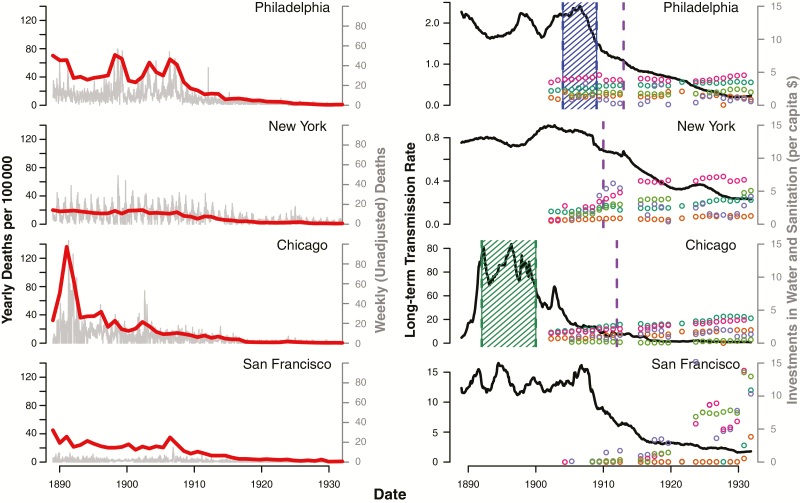
Observed time series and long-term typhoid transmission rates with water supply interventions and yearly investments in water supply systems are shown for each of the 4 cities. In the plots on the left, the observed time series of weekly deaths reportedly due to typhoid (gray lines) and the yearly typhoid deaths per 100 000 population (red lines) is shown for each city from 1889 through 1931. In the plots on the right, the estimated long-term typhoid transmission (gray lines), single water supply system interventions or events (dashed vertical lines: red denotes filtration, diagonal blue lines denote chlorination, diagonal green lines denote reversal of water flow, and purple dashed lines denote other intervention/event), and yearly water supply system investments (colored dots: dark green denotes receipts, orange denotes expenses, purple denotes outlays, pink denotes total value divided by 10, and light green denotes funded debt divided by 10) are shown for each city.

Despite being <100 miles apart, the levels and patterns of typhoid mortality and transmission in Philadelphia and New York were very different (Figure 2). Philadelphia, which averaged 43.1 typhoid deaths per 100 000 people per year from 1889 to 1910, drew its water supply from the Schuykill and Delaware rivers. These rivers, which traversed the city, were heavily contaminated with raw sewage from the >1000 miles of sewers that emptied directly into the rivers and their tributaries [50]. Various sand and gravel filtering stations were constructed throughout the city, beginning with the wealthier Roxborough and Belmont districts in 1904 and 1906, respectively, followed by the Torresdale district in 1907 and the remaining districts in 1909. Epidemics of typhoid fever in 1906 and 1907 were largely confined to the parts of the city that were still receiving unfiltered water [51]. There was a steady increase in water supply receipts (reflecting the increased cost and number of people served by the public water supply) and funded debt for the water supply and sewer system over this time period, which were inversely associated with the estimated long-term typhoid transmission rate [48].

New York City, on the other hand, drew its water from rural watersheds and constructed aqueducts to carry it into the city [52]. Typhoid mortality at the turn of the century was considerably lower than in Philadelphia (averaging only 22.4 typhoid deaths per 100 000 per year) and exhibited pronounced seasonal peaks in the late summer/early fall (Figure 2). The New York State Public Health Law of 1905 gave the city eminent domain to regulate land use in the upstate watershed region [53]. Chlorination of the water supply began in 1910, but filtration was only recently introduced in 2013. The water supply was expanded beyond the original Croton watershed to include additional reservoirs in the Catskills region between 1915 and 1928. Over this time period, typhoid mortality declined steadily. The estimated transmission rate of typhoid was inversely associated with water supply receipts, expenses, and funded debt and total value of the water supply system [48].

Chicago relied on Lake Michigan for its water supply, but the city’s sewage system drained directly into the lake via the Chicago River [54]. As a result, the typhoid mortality rate was 33.7 per 100 000 per year, and the city suffered from repeated outbreaks of cholera and typhoid in the late 19th century (Figure 2). In 1900, the Chicago Sanitary and Ship Canal was completed, which reversed the flow of the Chicago River so that sewage from the river would no longer empty into Lake Michigan [54]. Chlorination was introduced in 1912. Not only was Chicago the only city in the study to have statistically significant relationships between its typhoid transmission and all long-term investments in the water supply and sewer system, it also consistently had some of the strongest associations. For example, each $1 per-capita increase in water supply expenses was associated with an 82% (95% confidence interval, 59%–92%) reduction in typhoid transmission [48].

In San Francisco, water supply and sewer systems were mostly privately owned. Water was obtained from wells, creeks, and springs owned and operated under the monopoly of the Spring Valley Water Company [55]. The company was more interested in profits than public health, and as a result, the typhoid mortality rate was 23.7 per 100 000 per year (Figure 2). Following the 1906 earthquake and unsystematic rebuilding of municipal sanitary and water infrastructures that ensued, political will to publicly acquire the water supply mounted, and the city purchased the Spring Valley Water Company for $41 million in 1930 [55]. Municipal expenditures on the water supply spiked in 1930, and thus we found relatively weak and inconsistent associations between typhoid transmission and infrastructure investments in San Francisco [56].

Despite the significant variations of municipal sanitary, environmental, and technological interventions and their timing, the same concept emerges: increased and regular spending on sanitation systems was correlated with decreased typhoid transmission across US cities (Figure 2). In Britain and the United States, cities’ ability to take on debt was one of the most significant factors affecting municipalities’ ability to control enteric infections like typhoid [27]. It was expensive to build and maintain water and sanitation systems, so cities sought public ownership of the infrastructures, which allowed for municipal debt obligations. Most cities used a mix of debt, loans, and bonds to fund their urban infrastructure. From 1860 to 1922, US municipal debt increased from $200 million to more than $3 billion—a similar story emerges with regards to local government expenditure and municipal debt in Britain [23, 25, 41]. However, it was not just the initial outlay of money that mattered, but municipalities’ ability to maintain sanitary infrastructures and services consistently that had the most significant impact on public health, as reflected by the strong associations between financial investments in the operation and maintenance of water systems and declining typhoid transmission rates. Rather than “heroic” one-off investments in grand infrastructure schemes, the emergence of functioning municipal administrations that had money to maintain infrastructures and provide affordable services consistently across all segments of the population was essential for achieving sustained reductions in typhoid mortality. In the long term, enabling effective municipal sanitary and water service provision via sustainable credit schemes may be just as important as providing one-time funds for infrastructure investment.

## CONCLUSIONS

History offers a rich seam of data with which to evaluate various public health interventions. By fusing historiographic and epidemiological methodologies, we have come to 2 interrelated insights regarding the success of different typhoid interventions: (1) Effective sanitary infrastructures in Oxford and major US cities evolved not as a top-down and linear process of reforms but as a bottom-up response to local disease problems, social contexts, and power struggles in the relative absence of central government oversight; (2) in contrast to popular accounts’ emphasis on heroic moments of scientific and technological intervention, sustained financing of affordable water and sanitary infrastructures emerges as a crucial factor underpinning mid- and long-term disease control. In both Oxford and the United States, municipalities’ ability to take on affordable debt (via bonds, commercial loans, or long-term government loans) incentivized the formation of local coalitions around sanitary reform, often well before national laws mandated clean water and waste water disposal. This account of locally tailored, nonlinear solutions, and cheap municipal debt provides important insights for the control of typhoid in the rapidly urbanizing cities of the 21st century.

While the top-down health planning favored by international planners after 1945 could prove successful in nations with effective institutions and high levels of centralization, this policy approach often failed in areas characterized by high degrees of regional autonomy, weak government control, or lacking resources [57]. In 2005, the United Nations Millennium Task Force on Sanitation called for stronger institutions and better financing of sanitation for the approximately 2.5 billion humans without access to “improved” sanitation. New forms of debt-financed sanitary intervention include subsidized credit through bank guarantees and support for microfinance providers, alongside leveraging tools such as subsidies, grants, and tax breaks. These arrangements are directed at multiple societal levels, from households and communities to national governments as well as nongovernmental organizations and private sector companies [58]. In a way, many of the sanitary initiatives currently under way thus mirror 19th-century efforts to strengthen municipal infrastructures—but with a greater variety of financial tools and levels to target. As our historical examples show, expanding this approach of providing long-term financial support for local actor coalitions and for tailored locally owned municipal infrastructure development and maintenance may well prove a robust and affordable strategy for sustainable typhoid control.

## Notes

***Acknowledgments.*** The authors thank Andrew Pollard, Christoph Gradmann, and James Meiring for their insightful comments on this manuscript. Participants of our Higher Education Funding Council for England Global Challenges Research Fund (HEFCE GCRF) sponsored 2018 International Workshop, “Typhoid Past and Present,” gave helpful feedback on early versions of this manuscript, and the New Venture Fund has sponsored open access publication of this article.

***Financial support.*** This work was supported by funding from the Bill & Melinda Gates Foundation (grant numbers OPP1116967 and OPP1151153); the Wellcome Trust (strategic award 106158/Z/14/Z); the Higher Education Funding Council for England Global Challenges Research Fund; and the New Venture Fund.

***Supplement sponsorship.*** This supplement has been funded by the New Venture Fund.

***Potential conflicts of interest.*** V. E. P. received reimbursement from Merck for travel expenses unrelated to the subject of this article. All other authors have no conflicts of interest. All authors have submitted the ICMJE Form for Disclosure of Potential Conflicts of Interest. Conflicts that the editors consider relevant to the content of the manuscript have been disclosed.
